# Editorial: Population genetics and conservation of aquatic species

**DOI:** 10.3389/fgene.2022.1052740

**Published:** 2023-01-04

**Authors:** Shaokui Yi, Cong Zeng, Yanhe Li, Narongrit Muangmai

**Affiliations:** ^1^ School of Life Sciences, Huzhou University, Huzhou, China; ^2^ School of Oceanography, Shanghai Jiaotong University, Shanghai, China; ^3^ College of Fisheries, Huazhong Agricultural University, Wuhan, China; ^4^ Faculty of Fisheries, Kasetsart University, Bangkok, Thailand

**Keywords:** aquatic species, population, genetics, conservation, structure

In recent decades, efforts to protect many terrestrial taxa have slowed their rates of extinction. Unfortunately, the outlook for a number of aquatic organisms, such as amphibians, corals, fish and other aquatic species is still not optimistic. Many aquatic species are highly threatened by anthropogenic and environmental disturbances, such as climatic change, overfishing, habitat elimination and fragmentation, and invasive species (Buchanan et al., 2016; Sowińska-Świerkosz and Kolejko, 2019). However, the conservation of these species requires knowledge of their spatial diversity and population structure, and the inaccessibility of aquatic animals poses a great challenge to traditional surveys. Population genetics provides the tools to describe genetic diversity within and among populations, while it also provides the basic theory for understanding the evolutionary change and resulting patterns of genetic variation in different populations. This information greatly contributes to the integrated concept of biodiversity conservation, which is needed to define the goals and methods of conservation programs and to set priorities (Loeschcke et al., 2013). Understanding the genetic landscape of natural populations is one of the key concerns for the development of conservation management strategies. Meanwhile, population genetic data for economically important aquatic species is also essential for the optimal utilization of this genetic resource in breeding programs. Importantly, with the great advances of sequencing technologies, detecting genomic variation (e.g., microsatellites, mitochondrial genes and single nucleotide polymorphisms) is becoming increasingly inexpensive and efficient. Molecular markers have been extensively applied in population genetics studies of aquatic animals during the past decade (Chapman et al., 2012; Yi et al., 2019). A large number of molecular markers can provide an efficient means to infer the population history and status of examined species and to predict future changes. The number and type of markers used are critical factors when planning a population genetic study. In some cases, the results generated with different traditional markers, such as mitochondrial genes and nuclear microsatellite markers (simple sequence repeats, SSRs), have been inconsistent (Baisvar et al., 2018; Wang et al., 2019; Zhong et al., 2019). With the aid of cost-effective genotyping technology, genome-wide single nucleotide polymorphism (SNP) markers could help us to obtain more reliable population genetic data, which is of great importance to complement or replace existing conservation strategies. For this Research Topic, we gathered studies of aquatic populations that use these powerful molecular markers to interpret population structure, phylogeography, or evolutionary processes. Their findings can be directly applied to conservation efforts. The outcomes of studies could directly provide suggestions or implements for conservation.

Thanks to the combined effort of all the Editors, we are pleased to present 11 papers authored by 80 excellent researchers from various fields. The paper by Huo et al. focuses on the genetic diversity and population structure of *Triplophysa tenuis*, an important indigenous fish in the Xinjiang Tarim River that is facing overfishing and habitat degradation. For the study, a large number of SNPs were obtained with the genotyping-by-sequencing (GBS) method, and the eight populations were found to have high levels of genetic diversity, with substantial genetic differentiation among populations. Hu et al. investigated the genetic differentiation of populations of a commercially important sleeper fish, *Odontobutis potamophilus*, using SNP markers, and suggest several conservation strategies in their report. The work by Repullés et al. evaluates the genetic structure and connectivity pattern of the endangered coral *Cladocora caespitosa* across its entire distribution range in the Mediterranean Sea. Their paper provides a better understanding of this endangered scleractinian coral, which allows for more informed conservation decisions. Most interestingly, Gilles et al. report massive introgressive hybridization between two distinct genera in Cyprinidae, and they suggest that the hybridization could generate a wide spectrum of hybrids that are a potential source of important evolutionary novelties. The paper by Collins et al. evaluates life history variation in the anadromous migration of *Oncorhynchus mykiss* using whole-genome resequencing, and report that a region on chromosome Omy12 may represent a minor effect gene. Also in this Research Topic, papers related to population genetics of several fish species are presented, including *Triplophysa robusta* (Zhong et al.), *Hippocampus erectus* (Luo et al.), *Gymnocypris przewalskii* (Fang et al.), *Coilia brachygnathus* (Zhai et al.), *Schizothorax biddulphi* (Nie et al.) and *Hemiculter leucisculus* (Gu et al.). In these studies, SNPs, SSRs or mitochondrial genes were applied to successfully identify patterns of genetic diversity, population structure and phylogeography. Their results should be very useful for assessing the population dynamics of these species and for developing future conservation strategies.

In conclusion, all of the papers in this Research Topic evaluate the population genetics of important aquatic species using molecular markers. The efforts of these researchers further the understanding of aquatic genetic resources and can help guide conservation and management strategies of aquatic species.
